# Long-term results of visual function in posterior uveal melanoma patients treated with I^125^ episcleral brachytherapy in a Spanish referral Ocular Oncology Unit

**DOI:** 10.5935/0004-2749.20210047

**Published:** 2021

**Authors:** Nélida Vicente, Maria Antonia Saornil, Ana Almaraz, María Fe Muñoz-Moreno

**Affiliations:** 1 Ophthalmology Department, Hospital General de Segovia, Segovia, Spain; 2 Intraocular Tumors Referral Unit National Health System, Hospital Clínico Universitario, Valladolid, Spain; 3 Epidemiology and Preventive Medicine Department, Valladolid University, Spain; 4 Statistics Unit, Hospital Clínico Universitario de Valladolid, Valladolid, Spain

**Keywords:** Melanoma, Uveal neoplasms, Iodine radioisotopes, Brachtherapy, Visual acuity, Melanoma, Neoplasias uveiais, Radioisótopos do iodo, Braquiterapia, Acuidade visual

## Abstract

**Purposes:**

We analyzed patient, tumor and dosimetric characteristics of subjects in a Spanish population diagnosed with uveal melanoma treated with iodine 125 (I^125^) episcleral brachytherapy, who presented with post-treatment loss of useful visual acuity and global evolution of visual acuity.

**Methods:**

A single historic observational cohort study was undertaken. Patients with uveal melanoma were recruited between September 1995 and June 2015. Clinical, tumor and dosimetric data collection and visual acuity evaluations were performed under everyday practice conditions based on a useful visual acuity >0.1 on the decimal scale. The baseline analysis was performed using descriptive and survival analyses according to Kaplan-Meier curves.

**Results:**

A total of 286 of the 665 patients diagnosed with uveal melanoma received episcleral brachytherapy, and 198 were included in the study. The mean follow-up time was 75.3 months (95% CI = 68.0-82.6). Patients with post-treatment useful visual acuity loss (n=94, 47%) presented the following characteristics: visual symptoms (n=80, p-value = 0.001); iris color (brown n=33, hazel green n=49, p-value = 0.047); Collaborative Ocular Melanoma Study size (medium n=80, p-value = 0.159); tumor, node, metastasis stage (T2: n=38, T3: n=38, p=0.012); shape (nodular n=67, mushroom-shaped n=26, p=0.001); posterior pole involvement (n=47, p=0.04); recurrence (n=10, p=0.001); and dose administered in the fovea, optic nerve and center of the eye (p<0.002). Using Kaplan-Meier analysis, the mean overall survival of useful visual acuity was 90.19 months, and the probability of preserving useful visual acuity was 66% for one year, 45% for five years and 33% for ten years.

**Conclusion:**

Patients most likely to present with visual acuity loss were those with the following profile: elderly males with dark irises who were diagnosed with visual symptoms and exhibited a medium/ large melanoma with a mushroom shape in the posterior pole (near the fovea and/or optic nerve). All patients treated with episcleral brachytherapy are likely to present with visual acuity loss, which is more pronounced in the first few years following treatment.

## Abstract (pt)

**Objetivo:**

Analisar características individuais, tu morais e dosimétricas de pacientes diagnosticados com melanoma uveal, tratados através de braquiterapia epiescleral com iodo-125 (I^125^), que apresentaram perda da acuidade visual útil após o tratamento e analisar a evolução global da acuidade visual em uma população da Espanha.

**Métodos:**

Este é um estudo observacional de coorte histórica considerando pacientes com melanoma uveal diagnosticados entre setembro de 1995 e junho de 2015. Foram coletados dados clínicos, tumorais e dosimétricos e medida a acuidade visual em condições de prática clínica diária, considerando uma acuidade visual útil superior a 0,1 na escala decimal. A análise de base foi efetuada por curvas Kaplan-Meier descritivas de sobrevivência.

**Resultados:**

Um total de 286 dos 665 pacientes diagnosticados com melanoma uveal recebeu braquiterapia epiescleral e 198 deles foram incluídos no estudo. O tempo médio de acompanhamento foi de 75,3 meses (IC 95%: 68,0-82,6). Os pacientes com perda da acuidade visual útil após o tratamento (n=94, 47%) apresentaram as seguintes características: sintomas visuais (n=80, p=0,001), cor da íris (castanha: n=33, castanho-esverdeada: n=49; p=0,047), tamanho de acordo com o Collaborative Ocular Melanoma Study (tamanho médio: n=80, p=0,159), tumor, nódulo, estágio de metástase (T2: n=38, T3: n=38, p=0,012), forma (nodular: n=67, em forma de cogumelo: n=26, p=0,001), envolvimento do polo posterior (n=47, p=0,04), recorrência (n=10, p=0,001) e dose administrada na fóvea, no nervo óptico e no centro do olho (p<0,002). Na análise de Kaplan-Meier, o tempo médio de sobrevivência geral da acuidade visual útil foi de 90,19 meses e a probabilidade de preservação da acuidade visual útil foi de 66% por um ano, 45% por 5 anos e 33% por 10 anos.

**Conclusão:**

O perfil de paciente com maior probabilidade de perda da acuidade visual útil é o de homem idoso com íris escura, diagnosticado com sintomas visuais e melanoma de tamanho médio a grande, em forma de cogumelo no polo posterior (próximo à fóvea, ao nervo óptico ou a ambos). Todos os pacientes tratados com braquiterapia epiescleral terão perda da acuidade visual, mais pronunciada nos primeiros anos após o tratamento.

## INTRODUCTION

Uveal melanoma (UM) is the most common primary intraocular malignant tumor occurring in adults. Although it is a rare pathology, with a low incidence ranging between 4.3 and 10.9 cases per million inhabitants, UM is a highly aggressive neoplasm. UM is the main primary intraocular pathology and can be fatal in adults, with a mortality rate of almost 50% in the 10-15 years following diagnosis, regardless of the study assessment criteria employed^(1-3)^.

In the 1960-70s, the prognosis of UM for visual function in the affected eye and the life of the patient were fairly poor. In more recent times, greater possibilities have been developed to conservatively treat the primary tumor and preserve the eyeball. Improving patient survival has not previously been possible since the disease is systemic, and must be treated independently from the primary tumor^(4)^. Yet, differences have been observed in the evolution of visual function. Episcleral brachytherapy (EB) is the most frequent form of conservative treatment used, and is the first choice of treatment for medium and small UMs. Iodine 125 (I^125^) is the most widely used radioisotope in this treatment^(5,6)^. The functionality of the organ after treatment, which is measured by visual acuity (VA), seems to be influenced by multiple factors that depend on the patient, tumor, and radiation administered. Thus, it is difficult to evaluate organ functionality after treatment, since this requires a specific and reproducible protocol to ensure validity. Extrapolation of results from previous studies for comparison is complicated, since the analyzed factors and VA measurements used vary between studies.

This study describes the characteristics of a group of patients who presented with UM, were treated with I^125^ EB, and lost useful VA over an 18-year period measured in real and reproducible conditions.

## METHODS

A single historic observational cohort study was conducted on patients diagnosed with UM and treated with EB using I^125^ as a radioactive source at the Intraocular Tumours Unit of the Clinical Hospital of Valladolid between September 1995 and June 2015.

**Inclusion and exclusion criteria**: Refer to table 1. The principles of the Declaration of Helsinki were followed.

**Table 1:** Inclusion and Exclusion Criteria

Inclusion criteria
1. Posterior UM located in the ciliary body or choroids
2.1^125^ used as the radioactive source
3. Collection of brachytherapy dosimetric data
4. An initial VA of >0.1 in the affected eye
5. A follow-up >3 months
6. Informed consent provided by the patient
**Exclusion criteria**
1. UM located in the iris
2. Ruthenium 60 used as the radioactive source
3. Patients without collection of dosimetric data from brachytherapy; or those
treated in another center
4. A follow-up <3 months

Useful VA was considered as a VA >0.1 for at least two consecutive check-ups. A VA of ≤0.1 was defined as non-functional VA. Patients who underwent enucleation were included in the non-functional group.

An extensive ocular examination was performed, including evaluation of best-corrected VA measured with a decimal scale system by an ophthalmologist from the unit following a standardized protocol, anterior pole biomicroscopy, an ocular fundus examination with retinography, B mode/vector A ultrasound and tumor measurements. If the tumor size could not be correctly assessed due to the presence of extensive retinal detachment and/or media opacity, nuclear magnetic resonance and/or computed tomography were used to rule out extraocular extension.

The diagnostic criteria for UM were the presence of an ophthalmological lesion and echographic characteristics (height >1 mm and base >5 mm) related to the disease.

Size classification was conducted using the Collaborative Ocular Melanoma Study (COMS)^(7)^.

Indications for EB were medium tumors (COMS, T2, T3), small tumors at follow-up with demonstrated growth, and exceptionally large tumors (T4) in cases where the patient rejected enucleation, or in monocular cases.

The treatment was conducted in accordance with the protocol established by the American (American Society of Brachytherapy)^(5)^ and Spanish (Spanish Retina-Vitreous Society)^(9)^ guidelines for UM, with a target dose of 85 Gy to the tumor apex and a safety margin of 2 mm. Treatment was planned using the dosimetry program BEBIG Plaque Simulator version 2.16 (BEBIG, Berlin, Germany) and contrasted with an independent method using dose calculation and treatment duration of TG-43 parameters and associated updates^(10)^.

### Data collection

The patient characteristics included in the initial examination were gender, age, and reason for diagnosis (routine check-up or presence of visual symptoms). Ocular data included the best-corrected VA and the iris color of the affected eye.

The tumor data included the location, size according to the COMS and TNM classifications, maximum tumor diameter and shape, anterior and posterior tumor margins, length, latitude and affectation of the posterior pole, macular involvement, tumor recurrence, and presence of extraocular and/or systemic extension.

Radiation plaque data included the radiation dose to the tumor apex, fovea, optic disk, lens and center of the eye and the radiation rate.

### Follow-up

Patients receiving brachytherapy were followed up at one, three, six, and twelve months (during the first year); every six months for the next five years; and once a year thereafter to assess potential complications and treatment efficacy. At these visits, the best-corrected VA was measured, and ultrasounds, fundus eye exams, and liver echography with liver function blood testing were performed.

### Data registry and statistical analysis

An ophthalmologist from the unit was responsible for filling in the questionnaire data. The data were registered in a Microsoft Access database specifically designed for UM in 1992. Statistical analyses were performed with the Statistical Package for Social Sciences software version 20.0 (SPSS, Chicago, IL, USA).

For descriptive analysis of the variables, frequency distributions were used to evaluate qualitative variables, whereas quantitative variables were measured with means and standard deviations. The associations among qualitative variables were analyzed with Pearson’s chi-squared test. Quantitative values were compared with either Student’s t-test or the Mann-Whitney U-test as appropriate. For the survival analysis, the non-parametric Kaplan-Meier method and mortality tables were used, and comparisons were performed using the log-rank test or the generalized Wilcoxon test.

The characteristics of 198 patients treated with I^125^ EB were analyzed to evaluate loss of useful VA since the time of diagnosis (VA was considered useful when >0.1 in at least two consecutive check-ups) and the overall survival of visual function.

## RESULTS

Of the 665 patients diagnosed with UM, 286 received brachytherapy as the first therapeutic option. Finally, 198 of these patients fulfilled the inclusion criteria for this study. The mean follow-up time was 75.3 months (95% CI = 68.0-82.6). The characteristics of the patients, tumors, and treatment for the 198 patients are shown in table 2.

**Table 2:** Characteristics of the cohort

	N = 198	%
Gender		
Male	92	46.5
Female	106	53.5
Age (years)		
<50	51	25.8
50-69	105	53
>70	42	21.2
Presentation		
Routine examination	51	26
Visual symptoms	145	74
Iris color		
Brown	75	38.5
Hazel green	88	45.1
Blue-gray	32	16.4
COMS classification		
Small	15	7.6
Medium	170	85.9
Large	13	6.6
TNM classification		
T1	52	26.3
T2	87	43.9
T3	57	28.8
T4		
Maximum diameter		
<10	2	1
10-16	70	35.4
>16	122	61.6
Tumor shape		
Nodular	6	3
Mushroom	36	18.2
Diffuse	2	1
Location		
Choroid	189	95.5
Ciliary body	9	4.5
Anterior tumor margin		
Anterior chamber	1	0.5
Ciliary body	17	8.6
Ora serrata to equator	78	39.4
Posterior to equator	102	51.5
Posterior tumor margin		
Ciliary body	1	0.5
Ora serrata to equator	7	3.5
Posterior to equator:	190	96
> 1 mm ON	170	85.9
< 1 mm ON	20	10.1
Longitude		
Temporal	147	74.2
Nasal	51	25.8
Latitude		
Superior	114	57.6
Inferior	84	42.4
Posterior pole involvement		
No	114	57.6
Yes	84	42,4
Macular involvement		
No	163	82.3
Yes	35	17.7
Tumor recurrence		
No	188	94.9
Yes	10	5.1
Extraocular extension		
No	196	99
Yes	2	1
Systemic extension	198	100
No		
Radiation dose at tumor apex (Gy) (88.92 ± 8.85)	79	39.9
<85	70	35.4
85.1-90.0	49	24.7
>90.0	49	24.7
Radiation dose to fovea (Gy) (47.07 ± 41.37)		
<40	112	56.6
40.0-79.9	55	27.8
80.0-149.9	26	13.1
>150.0	5	2.5
Radiation dose to optic nerve head (Gy) (36.76 ± 31.88)		
<30	98	49.5
30.0-49.9	59	29.8
50.0-74.9	28	14.1
>75	13	6.6
Radiation dose to center of the eye (Gy) (30.52 ± 15.54)		
<30	107	55.2
30.0-49.9	63	32.5
50.0-74.9	21	10.8
>75	7	1.5
Radiation dose to the center of lens (Gy) (19.10 ± 13.76)		
<12	80	40.4
12.0-15.9	20	10.1
16.0-23.9	44	22.2
>24.0	54	27.3
Dose rate at prescription point (cGy/hr) (80.13 ± 30.68)		
<55	74	37.4
55.0-69.9	12	6.1
70.0-84.9	15	7.6
>85.0	97	49
Plaque type		
COMS	144	72.7
ROPES	54	27.3
Plaque shape		
Round	172	86.9
Notched	26	13.1

COMS= Collaborative ocular melanoma study; TNM= Tumor, Nodes, and Metastasis staging system; ROPES (This is the plaque type name, it is not an abbreviation)

In terms of gender, melanomas were slightly more common in females (53.5%). Fifty-three per cent of the patients were between 50 and 69 years old, with a mean age of 58 years. Seventy-four per cent of melanomas were diagnosed based on the presence of visual symptoms. The predominant iris color was hazel green (45.1%), followed by brown (38.5%). The average tumor height was 5.47 (min 1.20, max 12.11, standard deviation 2.39). The tumors were predominantly medium-sized (COMS 85.9%), nodular (80.8%) and exhibited a maximum diameter between 10 and 16 mm (61.6%). The main location of the tumor was the choroid (95.5%). A total of 51.5% of the lesions had anterior margins in the post-equatorial zone. In most cases, the posterior margin was located in the post-equatorial region (96%); within this region, 85.9% of the tumors were more than 1 mm from the optic nerve. A total of 42.4% of the melanomas affected the posterior pole of the eye; central involvement of this region, macular involvement was present in 17.7% of cases.

The mean dose to the tumor apex was 88.9 Gy, where the mean doses administered to the critical structures of the eye were 47.07 Gy for the fovea and 36.7 Gy for the optic nerve. The mean rate of dose absorption was 80.13 cGy/h, and the mean duration of implantation was 126 hours. The plaque used was the COMS type in 72.7% of cases, and had a non-cleavage shape in 86.90% of cases.

**Figure 1:**
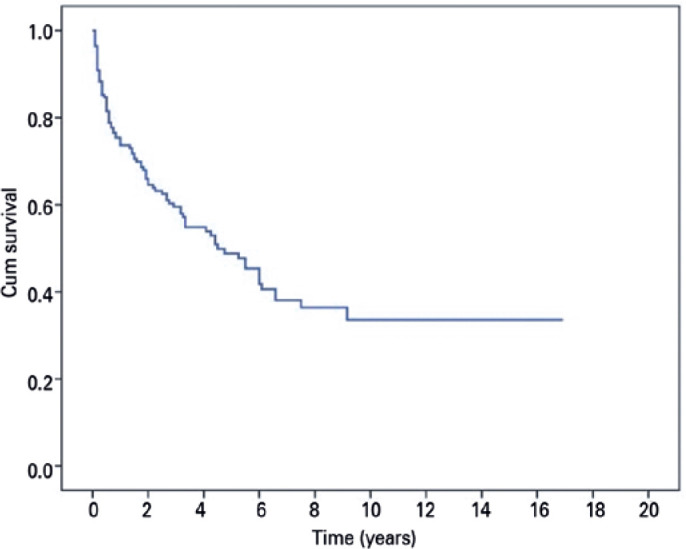
Overall survival of visual function Cum, cumulative.

The mean overall survival time of visual function was 90.1 months (95% CI = 75.0-105.3). The probability of maintaining useful VA was 66% at one year of treatment, 45% at five years, and 33% after ten years (Figure 1; Table 3).

**Table 3:** Overall survival of visual function

Year	0	1	2	3	4	5	6	7	8	9	10	11	12	13	14	15
**N**	198	129	97	78	60	**45**	38	27	19	14	8	7	6	4	4	4
**%**	75	66	59	54	48	**45**	37	36	36	33	33	33	33	33	33	33

A total of 94 of the 198 patients suffered loss of useful VA (Table 4). The variables presenting a statistically significant association with loss of useful VA were the reason for the diagnosis, iris color, TNM classification, tumor shape, and involvement of the posterior pole. Of the 145 patients treated with EB who presented visual symptoms at diagnosis, 80 (55.2%) suffered loss of vision. Of the 88 treated patients with a hazel green-colored iris, 49 (55.7%) lost useful VA. Of the 57 patients treated for T3 tumors, 38 (64.4%) reached a VA ≤0.1. Additionally, 26 (72.2%) of the 36 patients with mushroom-shaped tumors lost useful VA, 100% of the patients who received EB and presented tumor recurrence suffered vision impairment defined as VA ≤0.1, and 47 (56%) of the 84 subjects with involvement and treatment of the posterior pole lost useful VA.

**Table 4:** Proportion of cases with useful VA loss

	N=94/198	% loss of useful VA	p-value
Gender			
Male	48/92	52.2	0.217
Female	46/106	43.4	
Age (years)			
<50	27/51	52.9	0.631
50-69	47/105	44.8	
>70	20/42	47.6	
Presentation			0.001
Routine examination	14/51	27.5	
Visual symptoms	80/145	55.2	
Iris color			
Brown	33/75	44	0.047
Hazel green	49/88	55.7	
Blue-gray	10/32	31.3	
COMS classification			
Small	5/15	33.3	0.159
Medium	80/170	47.1	
Large	9/13	69.2	
TNM classification			
T1	18/52	34.6	0.012
T2	38/87	43.7	
T3	38/57	64.4	
T4	1/2	50	
Maximum diameter			
<10	28/70	40	0.220
10-16	62/122	50.8	
>16	4/6	66.7	
Tumor shape			
Nodular	67/160	41.9	0.001
Mushroom	26/36	72.2	
Location			0.175
Choroid	92/189	48.7	
Ciliary body	2/9	22.2	
Ciliary body	7/17	41.2	
Anterior tumor margin			0.671
Ora serrata to equator	40/78	51.3	
Posterior to equator	47/102	46.1	
Posterior tumor margin			0.257
Ciliary body	0/1	0	
Ora serrata to equator	3/7	42.9	
Posterior to equator > 1 mm ON	78/170	45.9	
Posterior to equator < 1 mm ON	13/20	65	
Longitude			
Temporal	67/147	45.6	0.364
Nasal	27/51	52.9	
Latitude			0.264
Superior	58/114	50.9	
Inferior	36/84	42.9	
Posterior pole involvement			0.04
No	47/114	41.2	
Yes	47/84	56	
Macular involvement			0.102
No	73/163	44.8	
Yes	21/35	60	
Tumor recurrence			
No	53/188	45.7	0.001
Yes	10/10	100	
Extraocular extension			
Yes	10/10	100	0.499
Systemic extension			
No	94/198	47.5	___
Radiation dose at tumor apex (Gy)			0.181
<85.0	38/79	48.1	
85.1-90.0	28/70	40	
>90.0	28/49	57.1	
Radiation dose to fovea (Gy)			0.002
<40	40/112	35.7	
40.0-79.9	34/55	61.8	
80.0-149.9	16/26	61.5	
>150.0	4/5	80	
Radiation dose to optic nerve head (Gy)			<0.001
<30	33/98	33.7	
30.0-49.9	31/59	52.5	
50.0-74.9	19/28	67.9	
>75	11/13	84.6	
Radiation dose to the center of lens (Gy)			0.686
<12	36/80	45	
12.0-15.9	8/20	40	
16.0-23.9	21/44	47.7	
>24.0	29/54	53.7	
Radiation dose to center of the eye			<0.001
(Gy)			
<30	38/107	35.5	
30.0-49.9	33/63	52.4	
50.0-74.9	18/21	85.7	
>75	5/7	7.14	
Dose rate at prescription point (cGy/hr)			0.068
<55	34/74	45.9	
55.0-69.9	10/12	83.3	
70.0-84.9	8/15	53.3	
>85.0	42/97	43.3	

COMS= Collaborative Ocular Melanoma Study; ON; TNM= Tumor, Nodes, and Metastasis staging system; VA= visual acuity.

The dosimetric variables which demonstrated a statistically significant association with loss of useful VA were the doses received by the fovea, the optic nerve, and the center of the eye. In these cases, a positive relationship was found between the magnitude of the dose and the patient’s probability of achieving non-functional VA.

## DISCUSSION

Of the 286 treated patients, only 198 met the inclusion criteria. These patients were chosen after excluding patients with a UM located in the iris, those treated with Ruthenium (Ru^106^), and those with a follow-up of <3 months, as these factors may have influenced VA progression. Firstly, patients with tumors located in the iris were excluded to avoid bias in the results, since these tumors have a low 5-year mortality (less than 3%). In addition, inclusion of these patients may create bias since the treatment directly affects the structures of the anterior side of the ocular globe, which may cause side effects and VA loss mechanisms different to those from posterior melanomas. Secondly, the exclusion of patients treated with Ru^106^ is justified since the difference in physical properties between the radioisotopes Ru^106^ and I^125^, creates a differing dose distribution in the different structures at risk. Thus, the subsequent complications for these structures, such the level of impact on VA, may differ. In addition, the number of patients treated with Ru^106^ was low, thus potentially resulting in bias and preventing a direct comparison. Thirdly, patients with a follow-up <3 months were excluded since this study aimed to investigate VA progression, and this follow-up may not have been long enough to obtain robust conclusions^(5)^.

Patient and tumor characteristics, as well as treatment approach, were evaluated in patients who received I^125^ EB and lost useful VA. These findings are important to understand the course of the disease and the visual prognosis of the patient, as well as the associated impacts on patients’ quality of life after treatment. However, analysis of VA is a particularly challenging objective, since it requires reproduction and external validation. As a result, few published studies have investigated the prognosis of post-treatment VA.

In terms of study group characteristics, the participants in this study varied from previous studies. Our study sample was very racially homogeneous, as all subjects were Caucasian. These results were in line with previous publications, which have found that the majority of UMs appear in Caucasian subjects (98%), and Caucasians are eight times more likely to suffer from this disease than African Americans^(11)^.

The Mediterranean population, including Spaniards, differs to the Anglo-Saxon and North American populations, as they generally exhibit darker hair, eyes, and skin than the latter groups, which may influence the prevalence of melanoma within the same Caucasian race. In our study, the highest incidence was found in women (53.5%). A similar result was noted in a study by Graell et al.^(15)^, which recorded cases of melanoma between 1994 and 2005 at the Bellvitge Hospital in Spain, and found 55% of the 303 participants with melanoma to be females. In addition, the percentage of females in a melanoma case series of 558 patients from Israel published between 1988 and 2008 by Frenkel et al.^(13)^ was 44.6%. Lastly, the COMS 16 study also demonstrated this trend, where the proportion of women with recorded cases of melanoma was 50.6%, compared with 49.4% for men.

The variation of iris color was mainly hazel green, followed by brown; which together formed a majority of dark, rather than clear, iris colors. These findings are supported by those previously published by Muiños et al.^(17)^. However, our findings contrast with other American and European case series, where the predominant color was blue-gray. Gallagher et al.^(18)^ and Guenel et al.^(19)^ concluded that the prevalence of melanoma in eyes with a clear iris was up to three times higher than that in eyes with a dark iris. Seddon et al.^(20)^ reported strong evidence that clear irises were a relative and independent risk factor for tumor development. A similar finding was reported by Weis et al.^(21)^ in a meta-analysis published in 2006. Further studies in the Mediterranean Caucasian population are necessary to confirm differences in the prevalence of iris color observed in our sample, since other publications are based on non-Mediterranean populations, in which the prevalence of subjects with clear irises was higher than that in Spain. This discrepancy may explain the difference in results between our study and other studies.

In our study, more than 95% of the tumors were located in the choroid, and diagnosis was made using visual symptoms in 74% of cases. This result was consistent with the size distribution found, where medium and large tumors represented between 73% and 93% of cases depending on the classification used, and were usually accompanied by visual symptomatology. The presence of visual symptoms is an important factor for diagnosis. Damato^(22)^ studied the frequency of symptoms based on the diagnosis and tumor sizes of 223 patients in the United Kingdom, where 55% of patients displayed visual symptoms at the time of diagnosis, and almost 80% of cases were medium/large tumors. The presence of symptoms was found to be directly related to the tumor size^(22)^. Esquelin and Kivelä^(23)^ published a study of 184 cases, in which 87% were diagnosed according to the presence of visual symptoms, and most were medium-sized tumors. In terms of the presence of visual symptoms, the results from our study lay between those of the two previous studies, and all cases demonstrated a clear relationship between visual symptomatology and tumor size.

The size distributions of tumors according to COMS and TNM were similar to those found in previous studies^(13,15)^. The classification with the highest prediction accuracy for the disease prognosis was developed by McLean based on the maximum tumor diameter^(24)^. For this reason, we included this classification in our study, and found that 61.6% of tumors had a maximum tumor size of between 10 and 16 mm. However, we were not able to compare this result with any other publication, since this classification has not yet been used elsewhere. Other factors related to the loss of useful VA, such as a mushroom shape, post-equatorial anterior tumor margin, post-equatorial posterior tumor margin, and macular involvement, were similar to previous studies, thus providing additional support for the validity of our work^(25,26)^.

In terms of dosimetric characteristics, the patients of this study were subjected to significantly lower doses administered in two critical structures of the eye (the fovea and the optic nerve) compared to patients in the COMS 16 study^(26)^ and the study by Shields et al.^(27)^.

According to these data, it was expected that using a tumor apex dose similar to that of COMS but with lower doses in key adjacent vision structures in our study would provide better results regarding the functionality of the eye compared to those of other publications. However, this was not the case, and therefore, it is possible that the impact of radiation on visual prognosis is not the only significant contributing factor, where multiple variables may be involved.

Most of the patients in our study presented VA loss, with half of the patients reaching a VA value equivalent to legal blindness in Spain (VA ≤0.1). It is difficult to compare results of other publications, since the final VA is highly dependent upon certain factors that are differ between studies. According to Char et al.^(27)^, who measured the rate of VA loss, a greater loss of VA was recorded immediately after treatment, and good VA three years after treatment was an excellent predictor of visual prognosis^(27)^. These findings differ from those of Gragoudas et al.^(28)^ published three years later, who found that two-thirds of patients with a VA ≥20/100 experienced a decrease in VA five years post-treatment, where the rate of decrease remained quite stable, in the range of 15 to 32%^(28)^. On the other hand, the COMS 16 study based the measurement of VA on the specific Bailey-Lovie optotypes protocol. Thus, the data from this study are not comparable with other publications, where VA was measured in a routine clinical check-up, or with the findings obtained from a statistical summary using VA from the last consultation, since patients with long follow-ups have been founde to present with fluctuations in vision^(29)^.

In our study, patients most likely to present VA loss were those with the following profile: elderly males with dark irises who were diagnosed with visual symptoms and exhibited a medium/large melanoma with a mushroom shape in the posterior pole (near the fovea and/or optic nerve). Patients over 70 years old had higher rates of useful VA loss, with a possible explanation being that tumors in patients at more advanced ages display more aggressive behaviors, although there is no scientific evidence to substantiate this.

The greater loss of VA observed in patients with dark irises may result from larger numbers of brown and green irises than other colors being investigated in previous studies. We found dark iris color to be a negative prognostic factor for the preservation of useful VA. However, we could not compare these results to other studies, as there are no publications investigating the implication and importance of iris color in VA loss after EB.

In the COMS 16 study, the patient exhibiting VA loss was a male with a medium-large, mushroom-shaped tumor close to the fovea and optic nerve^(25)^. According to the study by Shields et al.^(27)^, those most likely to lose useful VA after treatment were elderly patients with recurrent, medium-large, mushroom-shaped tumors in a location similar to that in the COMS study. Comparing these studies with our case series, all results regarding the significance of the patient’s age, as well as the tumor size, shape and location, are consistent. The present study may expand the parameters for improving a wrong visual prognosis, such as diagnosis using the presence of visual symptoms and a green or brown iris color, as opposed to blue.

In terms of dosimetric characteristics, the statistically significant parameters found in our study were the doses administered to the fovea, the optic nerve and the center of the eye. The overall dose was calculated to be the minimum received at these critical vision structures necessary to avoid negative effects on the final VA. The results obtained in this group indicated that the higher the dose received, the greater the percentage of patients who lost VA. On the other hand, the COMS 16 study only evaluated the doses administered to the fovea and optic nerve, and not to the center of the eye. Although the doses recorded were higher than those of the present study, the percentage of patients with useful VA loss and greater vision loss grew as the dose increased^(25)^.

Our study featured various strengths. This was a prospective study of 665 patients diagnosed with UM, of whom 198 were treated with I^125^ EB. Our study included one of the largest patient samples among studies investigating the progression of VA, both in our country and in Europe. The mean follow-up of 75.3 months internally confirmed the results at five years. The high number of patients treated and followed increased the odds of detecting statistically or clinically significant differences. The objective was to measure VA in everyday practice (i.e., in real conditions), which was hoped to increase reproducibility of results and avoid bias, since many studies feature strict ideal characteristics and thus provide results that are not highly comparable.

Despite the lack of uniformity in previous studies, the results of our study and those of other studies provide a common final conclusion. Overall, the patient is likely to experience irreversible vision loss after EB, which is more pronounced in the first few years following treatment; although this may vary considerably from one patient to another depending on their particular characteristics, which may predict the extent and rate of vision loss.

In conclusion, the majority of patients with UM treated with EB suffer a loss of long-term visual function that is greater in the first years following treatment. The profile of a patient most likely to lose VA is an elderly male with a dark iris who is diagnosed with visual symptoms and exhibits a medium/large melanoma with a mushroom shape in the posterior pole (near the fovea and/or optic nerve).

The results of this study require further investigation in order to confirm their association with loss of VA, and to detect factors that act as negative modifiable parameters on the final result. Additionally, the creation of a vision prognostic tool to be used before EB, based on the variables presented here, would be very useful and would contribute to estimation of final VA in each patient following treatment.
